# Immobility beyond borders: Differential inclusion and the impact of the COVID-19 border closures

**DOI:** 10.1177/02633957231173375

**Published:** 2023-05-17

**Authors:** Hannah Pool

**Affiliations:** Max Planck Institute for the Study of Societies, Germany

**Keywords:** borders, Covid, differential inclusion, migration, mobilities

## Abstract

This article discusses differential inclusion as it relates to mobility in Europe through migrants’ experiences of the closure of the European Union (EU) Schengen borders during the COVID-19 pandemic. Based on 36 comparative online interviews with three groups of migrants – Erasmus students, asylum seekers and seasonal workers – the article empirically investigates how differential inclusion is reflected in migrants’ perceptions of border closures and the impact of border closures on international mobility. Drawing on the concept of differential inclusion, I examine the divergent border mobilities in a moment of crisis. In the interviews, migrants’ reflections on borders are informed either by their own perception of borders, their surprise at the lack of awareness of borders for other migrants, or the realisation that closed borders are crossed for capitalist economic demands under high health risks. Taking this as its basis, the article makes two arguments. First, that preexisting differential inclusion exacerbated during border closures in a global health emergency. Second, that borders are not concrete but flexible in (im)mobilising people according to capitalist economic demands. In this way, the article contributes to an understanding of the process of rebordering that took place during COVID-19 and in which borders remained spaces of differentiation.

## Introduction

When the member states of the European Union (EU) individually decided to close their borders in March 2020 in response to the COVID-19 pandemic, these border closures did not manifest equally for all EU citizens and migrants and instead revealed the EU’s differing mobility regimes. The closures represented a break with the policy of free movement of people, services, capital and goods across the Schengen borders, which is considered as one of the EU’s most important achievements. However, this freedom of movement was already based on unequal mobility among different migrant groups, which became visible through the different treatment of their movements and increasing fortification of the EU’s external borders.

The article is a comparative case study of the border perspectives of three different groups of migrants moving across EU borders for education, legal protection and labour: Erasmus students, asylum seekers and seasonal workers. The empirical question the article aims to answer is: How does a moment of temporary border closure reveal the nature of differential inclusion by mobility in the EU? It answers this question by placing at its centre differing perceptions and experiences of this moment of border closure The article draws on interviews with three groups of migrants whose migration appears to be legally, economically and geographically very diverse. The border closure initially prevented all three groups from crossing borders. As conditions changed, however, they were either given the choice of whether to move, expected to move or immobilised further.

Based on this comparison, I argue that unequal mobility for economic imperatives in a capitalist system can be understood through differential inclusion. By providing an empirical comparative analysis of cross-border mobility in a specific instance of border closure, the article contributes to the academic discussion on the concept of differential inclusion.

The structure of the article is threefold: first, I introduce the literature on embodied borders, differential inclusion and pandemic-related border closures. Second, I describe the methodology and contextualise the semi-structured online interviews I conducted with 36 Erasmus students, asylum seekers, and seasonal workers and with 16 experts. Third, in the discussion I contextualise the informants’ statements on borders and analyse them through the concept of differential inclusion as imposed and exercised by borders. In doing so, I show how the groups reflected differently on their perceptions of borders and the implications of borders for their mobility practices during the COVID-19 pandemic. In the conclusion, I relate my findings to the existing literature and identify the potential for future research on borders and differential inclusion in Europe.

## Embodied borders, differential inclusion and COVID-19 border closures

In exploring the role of migrants’ differing (im)mobilities and perceptions of borders during the COVID-19 pandemic, the article draws on three strands of literature. The first is that of immobility regimes and embodied borders. Mobility has been central to migration studies (Larsen et al., 2006; Sheller and Urry, 2006; Urry, 2007). Calls have been made to extend this original focus on movement within migration studies to stasis in mobility regimes (Glick Schiller and Salazar, 2013). In this, it is borders which most clearly manifest and visualise immobilities (Khosravi, 2010, 2019). While the literature of border studies originally focused on the materiality of borders per se, associating state borders exclusively with the idea of a walled territory in an international system of Westphalian states, more recent research has turned to the idea of bordering processes and the construction of symbolic and conceptual boundaries through borders. The academic debate ranges from arguing for a world of borders, as Étienne Balibar (2002) has done, to the idea of a borderless world in globalised capitalism. Didier Bigo (2010) linked the idea of economic movement to an increase in security narratives by portraying borders both as sites of economic exchange and as military lines of security enactment. In light of the global increase in border fortifications between states, borders have once again featured prominently in migration studies. There is an extensive literature on the border fortifications of nation-states, focusing on the digitisation of borders (Latonero and Kift, 2018; Leese et al., 2022; Vukov and Sheller, 2013), the securitisation of migrants in border spaces (Scheel, 2013a) and the role of external agencies in border security (Vaughan-Williams, 2008).

Borders, however, are perceived in terms of their materiality but prove to be flexible, stretchable and malleable. This capacity for differentiation is the basis for an analysis of the differential treatment of movement by borders. Balibar argues that borders are constructed ‘not merely to give individuals from different social classes different experiences of the law [. . .] but actively to differentiate between individuals in terms of social class’ (Balibar, 2002: 81–82). This corresponds to the notion of embodied mobility originally developed by feminist geographers, which states that borders are regarded as subjective because they apply to and operate differently for different groups of migrants. Recognising that peoples’ mobility follows what Doreen Massey (1993) called the ‘power-geometry’ of politics and access of movement, Jennifer Hyndman goes further and frames mobility as being constituted by ‘economic, geopolitical, gendered and racialized relations’ (Hyndman, 2004: 169). Borders are thus embodied through class, gender and race (Hyndman, 2004; Luibhéid, 2006; Scheel, 2013b). The notion of embodied mobilities is crucial in that it allows an understanding of how borders subjectively determine, facilitate or impede the passage of different groups of migrants and thus shape how migrants perceive borders.

The second strand of literature on which the article draws therefore centres on the concept of differential inclusion, which emerged in critical migration studies in the early 2000s. Sandro Mezzadra and Brett Neilson theorise differential inclusion as ‘the production of multiple subject positions, as a device of exclusion’ (Mezzadra and Neilson, 2013: 268). Borders are recognised as sites of struggles in which not only the temporal and spatial framework of capitalism is structured, but the movement of capital and labour is also directed and controlled by and through bordering practices (Mezzadra and Neilson, 2013: 159). In this context, differential inclusion explores ‘how inclusion in a sphere, society or realm can involve various degrees of subordination, rule, discrimination, racism, disenfranchisement, exploitation and segmentation’ (Casas-Cortes et al., 2015: 79). Rather than viewing borders as mere instruments of exclusion, the literature on differential inclusion illustrates how migrants are selectively and deliberately included in certain rights of a host society (Andrijasevic, 2009; Mezzadra and Neilson, 2013). The exclusion of migrants who are regarded as ‘illegal’ is thereby a category within a system that aims to exploit these migrants (De Genova, 2002).

Illegalisation of migrants operationalises differential inclusion according to the imperatives of labour markets. The concept of differential inclusion has been used to explore how cross-border mobility affects academic elites, tied labour contracts and immobility differently through externalisation (Casas-Cortes et al., 2015). It has been applied to refugees (Baban et al., 2017) and migrants with precarious legal status (Fabini, 2017; Giglioli, 2019; Goldring and Landolt, 2022; Segrave, 2019), but less so to migrants with temporary status, such as seasonal workers. Here, I draw primarily on research that has used the concept of differential inclusion to compare different forms of cross-border mobility of migrants (Könönen, 2018; Vosko, 2022; Wotherspoon, 2018). By exploring perceptions of border regimes, I aim to contribute to the debate by examining continuities in a moment of crisis. The relevance of the concept of differential inclusion lies in its ability to illuminate the tense relationship between inclusion and the various forms, modes and privileges in the autonomy of migration. The literature on the autonomy of migration focuses on the ‘subjective movement’ of people in relation to the conflicts that arise from unequal mobilities (Mezzadra, 2011; Scheel, 2019). In this article, three different cross-border mobilities are analysed to understand the differential inclusion occurring at a particular moment of official general border closure during a declared pandemic crisis.

The third strand of literature looks at border closures during a pandemic, with a regional focus on the EU Schengen area. There is a long history of pandemics and government decisions to close their borders in response to them. Christian Enemark (2009) illustrates how border closures during pandemics are perceived as protection against security threats. The border closures between Spain and France in 1822 because of yellow fever and those in West Africa because of Ebola virus, meanwhile, were events of rebordering due to a fear of pandemics (Radil et al., 2020). Within Europe, border closures in response to COVID-19 in March 2020 contradict the idea of a borderless Schengen area that allows the free movement of goods, services, people and capital. The decision by national governments to close Schengen borders due to health concerns over the spread of Covid reshaped mobility within the EU (Carrera and Luk, 2020). The border closures and subsequent reintroduction of internal controls further held symbolic capital in the EU (Thym and Bornemann, 2020). The understanding of solidarity within the EU has thus also been shaped by the closure and reopening of the Schengen borders (Cicchi et al., 2020). The profound consequences of the closures affected cross-border commuter traffic (Novotný, 2022), tourism (Seyfi et al., 2020) and migrant workers (Robin-Olivier, 2020). In the literature, the repercussions of border closures for individual groups of migrants has been examined, but there is a lack of comparison between them, and little consideration of how migrants’ perceptions of borders and cross-border mobilities have been shaped by this experience.

This article aims to bring together the three different strands of literature by comparing the border perceptions of three migrant groups in order to analyse how differential inclusion was exacerbated during the March 2020 EU Schengen border closures due to the COVID-19 pandemic.

## Data and method

The study is based on 36 semi-structured interviews I conducted with 12 people from each group: Erasmus students, asylum seekers and seasonal workers. The first group, Erasmus exchange students, cross borders to receive an international education. Since its inception in 1987, the EU-funded Erasmus programme has enabled more than 11.7 million students to study or intern at a range of partner universities (European Commission, 2021: 18). The 12 Erasmus students interviewed here are German, Greek, Romanian and Italian nationals and have studied at host institutions in Greece, Germany, Spain, Sweden and Finland.

The second group, asylum seekers, are people who cross borders into the EU to seek protection under the Geneva Refugee Convention and the Geneva Protocol. Between 2019 and 2020, the number of asylum seekers decreased from 698,000 to 471,000 (Eurostat, 2022). In addition, with the closure of borders, community shelters and asylum camps were placed under further confinement (Jauhiainen, 2020; Veizis, 2020). The asylum seekers interviewed here are Syrian, Afghan, Tajik and Iranian nationals residing in Germany or Greece.

The third group, migrant seasonal agricultural workers, move to a country other than their home country for a limited period of time to perform physical labour (OECD, 2001). It is estimated that 360,000 seasonal migrants are employed in Italy, 200,000 in France and 255,000 in Germany (Bundesministerium für Ernährung und Landwirtschaft, 2022; Mitaritonna and Ragot, 2020). Since most seasonal workers are EU citizens and therefore do not require work permits, there are no exact numbers. The migrant seasonal workers interviewed in this study are Romanian citizens working in Germany.

In addition to the 36 main interviews, 16 expert interviews were conducted with union workers, think tanks, social workers and legal advisors to contextualise the findings. All interviews took place between January and July 2021. Due to the pandemic, they were held either online via Zoom or a platform requested by the informants, or by telephone. The interviews lasted between 20 and 60 minutes on average and were conducted in German, English and Farsi. In the semi-structured interviews, key questions were asked about experiences during the COVID-19 pandemic and how these had an impact on the perception of borders, which led to follow-up questions to generate a free-flowing conversation (Roulston and Choi, 2018). The limitations of this interview practice lay in the extent of the data generated as well as the challenge that the data might be too divergent to be compared. However, the extensive coding process allowed the data to be clustered and theorised coherently. The data were analysed using MaxQDA through an inductive coding process in which the emerging in vivo codes were used to theorise the findings (Saldaña, 2013).

The interviewees were recruited using the snowball method (Atkinson and Flint, 2001), which involved asking respondents for referrals to other potential interviewees after each interview. The entry points to the individual groups were the chairperson of the Erasmus Student Network in Germany for the Erasmus students, a spokesperson of the asylum seekers community in Berlin for the asylum seekers, and the advocacy group Initiative Faire Landarbeit for the seasonal workers.

This form of qualitative research has some limitations. As a German researcher, I was able to conduct the interviews in German, English and Farsi only. While access to the group of Erasmus students was relatively easy due to our common educational background, and to the group of asylum seekers due to a certain standing based on my previous research with the communities, I had to rely heavily on social workers and advocacy groups as gatekeepers to access the seasonal workers.

While none of the Erasmus students and asylum seekers I approached declined my interview request, 6 of the seasonal workers I contacted refused to be interviewed, and of the 12 who did agree to be interviewed, all refused to be recorded. This points to the high level of dependency and hierarchies for seasonal workers in the German agricultural context.

## Background: Differing (im)mobilities in the EU during the COVID-19 border closures

The difference in (im)mobilities across borders within the EU has long been discussed, and it became particularly visible during the COVID-19 pandemic. To put this in a legal context, in Article 3(2) of the EU Treaty, the EU promises ‘to offer its citizens an area [. . .] without internal frontiers’. In this context, the European Single Market provides for the four freedoms: the movement of goods, the movement of capital, the establishment and provision of services, and the movement of people. The Citizens’ Rights Directive 2004/38/EC grants EU citizens the freedom to travel, reside and work in another EU country. Within the 22 Schengen states, it also provides for the abolition of passport controls as the main feature of mobility within the EU. In 1992, the Maastricht Treaty introduced a form of EU citizenship that ‘derived from holding citizenship in a member state and thereby complementing, rather than replacing national citizenship’ (Geddes, 2000: 101). Scholars in the field of migration and border studies have nevertheless pointed to the differentiation that border policies can create. Jef Huysmans (2004) argued that the idea of freedom in the Schengen area increasingly securitises the free movement of people, especially in the context of long-term migration and asylum. This became particularly visible with the reintroduction of Schengen border controls in 2015 to curb and control the passage of asylum seekers within the EU (De Genova, 2017).

The outbreak of the COVID-19 pandemic in March 2020 further demonstrated these unequal mobilities. This can be exemplified by the entry requirements for Germany, which varied widely for the three groups studied. Following the World Health Organisation’s classification of COVID-19 as a pandemic, the European Commission (EC) recommended temporary restrictions on nonessential travel from third countries on 16 March 2020, with which the German government complied. The EC defined this regulatory framework in more detail on 30 March in its Guidelines on Travel Restrictions (2020/C102 I/03). These Guidelines introduced differentiation between migrant groups by exempting seasonal agricultural workers because they were categorised as performing an essential function. When the border closure did affect seasonal workers, German agricultural advocacy groups lobbied for special border opening that would allow 40,000 seasonal workers to cross into Germany between 2 April and 30 May 2020 (Initiative Faire Landarbeit, 2020: 5). As a result of the pandemic, working conditions deteriorated under high epidemiological health risks and longer working hours to compensate for the overall shortage of workers (Neef, 2020; Weisskircher, 2021).

At the same time, asylum seekers were denied border crossings, despite the will to provide international protection, and the resettlement programme was halted, asylum procedures were postponed and integration programmes put on hold (Kluge et al., 2020; Kondilis et al., 2021). The border closures moreover placed community shelters and asylum camps under further confinement (Jauhiainen, 2020; Veizis, 2020).

Erasmus students, meanwhile, had the privilege of being able to choose individually whether to return to their home country or remain in their host country. In a survey of 20,000 Erasmus students in March 2020, 40% said they would return to their home country immediately, while 43% initially chose to stay in their host country (Erasmus Student Network, 2020: 26). With the digitisation of teaching, physical presence in class has become detached from academic study, allowing transnational education to continue from an (individually) chosen location.

These legal frames reflect the differing regulation of (im)mobility at the point of entry to a country during a time of crisis. The following section discusses the coded interview segments with the three groups of migrants and considers them in the context of differential inclusion.

## Discussion: Border perspectives and differential inclusion

I now turn to the empirical data to illustrate how differential inclusion was reflected in the way Erasmus students, asylum seekers and seasonal workers perceived borders and assessed their mobility during the March 2020 border closure. Migrants’ perceptions and crossings of borders are at the centre of the analysis, since in migration ‘[m]odes of entry represent a primary mean through which differential inclusion’ (Vosko, 2022: 131) occurs. My analysis is organised around two aspects of border (im)mobilities that were recurrent themes in the interviews: first, the perception of borders; and second, the (in)ability to cross borders during the COVID-19 closures. The perception of borders is subjective and depends on the ease with which they can be crossed: while for Erasmus students borders only became visible when they were closed, asylum seekers reflected on the differential inclusion enacted by borders in relation to other migrants; for seasonal workers, the border remained a place of crossing for economic imperatives.

### Erasmus students: Aware of the border for the first time

For all the interviewed Erasmus students, who held German, Italian, Greek or Romanian passports, the border closure meant a rupture at which they perceived the existence and consequences of the Schengen borders for the first time. When asked if he experiences the EU borders in his everyday life, Simon, a German Erasmus student who was in Greece during the pandemic, immediately responded:

Almost not at all. Now, with Corona, some borders were closed and you couldn’t just cross them easily. For the first time, it became much more obvious that with a German passport I can enter almost everywhere without any problem. Especially to EU countries, of course, but even outside the EU, it is possible with a German passport [to enter a country]. That’s why I didn’t really notice borders because it’s not really an obstacle I would have to overcome, but through Corona, I have a different view of what state borders can mean to other people.

Simon’s answer was representative of all the Erasmus students’ interview statements that the pandemic changed their perception of borders. In his statement, Simon creates a temporal separation between a time before and after Covid and illustrates how this determined his perception of border closures. By stating that he could go ‘almost everywhere’, he underlines his perceived unlimited mobility; to him, the few restrictions imposed at the time underscored the extent of his freedom of movement. His answer presupposes that border privileges must be reflected upon, but he was only able to do so when faced with a changing environment. At the same time, Simon refers to the experiences of other people, who – it is assumed – do not have the same passport and thus mobility privileges that he has become aware of and enjoys. His experiences coincide with those of the other Erasmus students interviewed, with 10 out of 12 saying that they had not been conscious of the Schengen borders before the pandemic. This is further illustrated by Mona, a German Erasmus student in Spain, who described the border closure as a breach of her perceived freedom of movement within Europe:

I increasingly followed the news from different countries, including air travel, and there were more and more restrictions, which of course worried me. [. . .] It became a bit clearer to me that we actually have a lot of freedom in Europe, to be able to travel across different countries without any borders, without standing in front of borders and being controlled. And that’s only really happening again now because of Corona and I somehow never really noticed that before. For me, Europe was more associated with freedom.

The ability to ‘overlook’ borders is an expression of the privileged position that EU citizens could enjoy when crossing borders within the Schengen area without border controls. The Erasmus students interviewed were all in their early twenties (born between 1998 and 2003) and therefore had no memory of previous border posts in the Schengen area. The way Mona refers to ‘we [. . .] in Europe’ suggests that she is generalising her perception of borders to other Europeans, who presumably did not notice borders in Europe before either. She only personalises this in the last sentence, when she makes her own connection between travelling and freedom.

Given their legal, academically privileged and EU-funded mobility, the Erasmus students interviewed mostly only reflected on their embodied mobility (Hyndman, 2004) at the moment of mobility disruption during the pandemic. Border controls are subliminally something that only others, not those in Europe, experience. The living reality of asylum seekers or seasonal workers in Europe is hereby excluded.

### Asylum seekers: Recognising and comparing closed borders

The asylum seekers I interviewed were well aware of the existence of borders, which they physically felt when they crossed undocumented to seek asylum on EU territory. The border closure due to COVID-19 was less tangible in this context, as it had no direct impact on the already closed borders they had personally experienced. However, in order to contextualise and make sense of the border closures, they also contrasted their experience with that of other migrants. They stressed that they were aware that the closure of the Schengen borders in March 2020 was a general disruption of the mobility regime which in the past had mainly affected and hindered their cross-border migration.

Bahman, a 40-year-old man from Iran who was staying in an asylum shelter in a major German city at the time of the interview, described how his migration trajectory made him feel the power of borders:

I know borders, Hannah. In my homeland, I understood very well what borders mean. You can say that only for people from Germany [the border closure in March 2020] was terrible. Now [in May 2021], you from Germany understand that borders mean borders. Borders are very important.

Like Simon, Bahman contrasts how his perception of borders differs from that of other people – those with EU passports. He divides perceptions of borders into countries where people perceive borders in their daily lives and those where borders were overlooked until the border closure during COVID-19 created a rupture. The way Bahman addresses me directly as a researcher indicates his need to also show me, as a German passport holder, that I too may not be aware of borders. The distinction he draws between people ‘who know borders’ and those who have to learn to ‘understand’ them persisted in his reflections throughout the interview. Bahman went on to give anecdotal evidence of the unreflective ways in which German passport holders overlooked borders when they travelled before the COVID-19 closures: ‘When I arrived in Germany, I saw that every three months at least, Germans travel to another country. That was very surprising for me. What is this? We cannot do that in my home country [Iran]’.

The sudden closure of the EU Schengen borders prompted many asylum seekers to reconsider and reflect on the closed borders they had faced when first entering the EU. Sarina, a 21-year-old student from Afghanistan, compared the closed borders in 2020 with her experience of arriving in the EU in 2018:

Suddenly, I felt very restricted [. . .] I also noticed that all other people felt confined, restricted, they weren’t allowed to enter the country and even couldn’t visit their family. Because of Corona, they all felt very alone and depressed. [. . .] When I came to Europe and to Germany, I noticed and saw the European borders. That was in Hungary, and there soldiers also closed the borders and it was very difficult for people to enter. That reminded me a bit of that moment.

By linking the 2020 border closure, which applied equally to everyone, to her personal experience at the EU external border in Hungary, Sarina categorises her immobility. She draws on the shared emotion – ‘they all felt very alone’ – to create a general circumstance that affected not only her as an asylum seeker but also everyone else. As Doreen Massey (1993) has argued through the power-geometry of mobility, those who are criminalised and displaced and whose movement is securitised often face restricted mobility as they are put on hold. This form of immobility is enforced through mobility regimes for people who seek asylum and are held in camps, border spaces or positions of waiting (Clifford Collard, 2021; Gill, 2009; Pettit and Ruijtenberg, 2019). What is new, however, is the transferral of this imposed asylum immobility to the circumstance of the global pandemic.

### Seasonal workers: Imposed mobility during the pandemic

While Erasmus students and asylum seekers described their perception of borders in the context of their immobility, migrant seasonal workers did not mention their perception of borders either prior to or during the COVID-19 border closure. Rather than being immobilised, they were continuously moved across borders during the pandemic: for migrant seasonal workers, cross-border mobility is linked to their physical labour and their economic contribution.

Alexandru Petrescu, a seasonal Romanian asparagus picker working in the German region of Beelitz, described in our interview that it was crucial for him to come to Germany during the pandemic to provide financially for his family. He stressed that his job depended on his mobility to get to Germany, and that the German farmer who employed him had provided him with information and logistical help to cross the closed EU borders. This experience was also shared by the other 11 interviewees, who also did not want their interviews to be recorded. In a recorded interview, Stephan Müller, a Romanian-speaking social worker for seasonal workers in Germany, expressed frustration over the simultaneously closed borders for general passage but open borders for physical labour in agriculture:

‘What’s the point of something like the European Union? That which is always invoked as unity, as values. And at the end of the day, borders can be opened and closed, just as economic forces dictate. But for ethical reasons, every nation-state is on its own again’.

As Alexandru and 23-year-old Luca, a strawberry picker, both emphasised, there is a separation over which Romanian workers were allowed to move based on their economic value to the German economy. Their and Stephan’s responses express an experienced hypocrisy of border mobility that determined who could and who could not cross borders during a pandemic based on the economic surplus a person generates in a capitalist system. The COVID-19 border closure reignited an older debate over hiring unemployed German citizens in seasonal agriculture (Holst et al., 2008), in that case whether they should be hired for the asparagus and strawberry harvests. However, it soon became apparent that this supposedly ‘low-skill’ work indeed required many skills that German unemployed people or students were not willing or equipped to provide (Initiative Faire Landarbeit, 2020; Seufert, 2020).

Mihai, a seasonal worker in his mid-30s, described the strain of not being able to return to his family in Romania throughout his work contract. Movement was only possible in one direction. More than 40,000 seasonal workers crossed the generally closed EU Schengen borders from Romania into Germany to work. However, those who were already in Germany were not allowed to move. Elena Stancu, a Romanian expert for seasonal workers, describes this conditional placement:

After the pandemic was declared in March, April, and May, in 2020, they [Romanian seasonal workers in Germany] were very worried about their children who are alone at home, because all these families have children at home who are taken care of by their grandparents, or relatives, I don’t know, an uncle or an aunt. And they felt helpless. Many of them told us: ‘We felt helpless. We were here. The children were in Romania. And we couldn’t help them. We didn’t know what was happening’.

Crucially, apart from the Romanian seasonal workers who were incentivised to cross borders into Germany to work, those who had already brought their physical labour to Germany as a host country that needed cheap and experienced agricultural labour were not allowed to return home. For them, the general border closure applied, and the privileged border crossing into and from the host country for seasonal workers was suspended. Elena Stancu continued: ‘If something happens you only have the right to work because there were special plans during the lockdown in Germany for Romanian workers. [. . .] But what if something happens at home?’ The differential inclusion of seasonal workers is reduced to their crossing the border as migrant workers. Other categories, such as being a family member or a care provider, are disregarded in this labour-extracting passage.

The way some seasonal workers describe their continuous cross-border mobility in a period of official border closure illustrates what Rutvica Andrijasevic sets out when arguing that transnational sovereignty is bound to the ‘transnational reorganization of labor that can only in part be regulated by the nation-state’ (Andrijasevic, 2009: 389). In this case, it was lobbying by the agricultural sector that enabled seasonal workers to move their labour and harvest the fields in another country during a global pandemic. The argument of seasonal demand for labour, coupled with a securitised language of food demand, was used to justify the request for workers. At this moment, borders became passable despite the general lockdown, illustrating how, as Andrijasevic writes, the state’s heightened security over its territory affected labour relations and working subjects.

### The differential inclusion of migrants

Differential inclusion provides insights into how border crossings are differentiated by citizenship, class, and labour value. Thus, in a moment of crisis, border perceptions can reveal the differential inclusion inherent in the EU mobility and border regime. The three interview segments in this study show how border awareness is a subjective dimension of differential inclusion. While Erasmus students could continue their education transnationally, asylum seekers were further confined, and seasonal workers were consciously moved at a moment of border closures. As argued in the literature on differential inclusion, the mobility of different groups of migrants is desired, encouraged or hindered differently. I therefore take the ways in which the processes and strategies to do so interact with subjective experiences and practices to examine interferences and disruptions (Mezzadra and Neilson, 2013: 133) are therefore taken to examine the individual perceptions of borders and border crossing practices during the closure of the Schengen borders. Focusing on the moment of immobility across borders makes it possible to examine how movement is redefined and enabled based on benefit to and as prescribed by the state.

This examination produces two insights. First, European borders separate not merely by citizenship but also by economic value. The European borders considered here, as Enrica Rigo observes, have an ‘ambivalent dimension of the frontier as a permeable area so as to reclaim the subjective role of those who come from “outside” and to understand their positions with regard to the polity’s boundaries’ (Rigo, 2005: 4–5). In the cases examined, border and citizenship regimes are intertwined in their exclusion of non-EU citizens, in this case asylum seekers, from cross-border mobility. However, during the pandemic, seasonal workers, who are EU citizens from Romania and Bulgaria, were incentivised to move across borders, not because of their EU citizenship but for their physical labour to generate an economic surplus. This illustrates how the labels ‘exchange students’, ‘refugees’, ‘seasonal workers’ and ‘citizens’ define the cross-border movement of people during the border closure.

Second, the act of perceiving borders is linked to privileged mobility. As outlined in the literature on differential inclusion and embodied mobility, borders are perceived differently depending on the purpose of crossing, legal protection and economic necessity. Erasmus students were unaware of borders that had not interfered with their mobility before the COVID-19 border closures, while asylum seekers who had to cross borders to enter the EU to claim asylum had already physically experienced borders. During the general border closure, however, the need for labour determined who could, or had to, cross borders. As Dimitris Papadopoulos and Vassilis Tsianos argue, the analytical focus should be on ‘the primacy of migrants’ mobility, that is to read capitalism through migration and to understand sovereignty through mobility, rather than the other way round’ (Papadopoulos and Tsianos, 2013: 184). The interview segments from the three groups examined here show that the European border regime is a system of differential inclusion in which the freedom of movement of some takes precedence over the freedom of movement of others. The concept of differential inclusion underlines what Casas-Cortes et al. (2015: 79) call the ‘*productive* aspects of the border’, where race, gender and class are interlinked and in conflict with each other when differentiating between border crossing by migrants with different reasons for migration – work, education or protection – as discussed in this article. The closure of borders during the COVID-19 pandemic put the Schengen borders at the centre of EU political life. However, it did so by redistributing privileged border crossings based on economic advantages and the generation of economic value under the heightened security risk of a pandemic crisis.

## Conclusion

In this article, I have examined differential inclusion as imposed by borders within the Schengen area by comparing how three different groups of migrants perceived borders and (im)mobility when the borders were officially closed to everyone in March 2020 due to the COVID-19 health security risks.

Through its empirical analysis, the article contributes a novel comparison between three different groups of migrants – Erasmus students, asylum seekers and seasonal workers – who cross borders for the purposes of education, labour and safety. Comparison of their perceptions of borders sheds light on how migrants make sense of borders and regulations, and how they assess the impact of borders on their mobility practices. During the border closures, migrants experienced forms of (im)mobilisation based on their economic contribution. While some migrants were moved to generate economic surplus despite heightened health concerns, others were immobilised further, and those who were already privileged were able to renegotiate their transnational spaces. With this empirical qualitative research situated in the literature on differential inclusion and embodied mobility, their perspectives make an important contribution to the concept of border perceptions. Thus, the phase of Schengen border closures in March 2020 revealed how the European border regime of differential inclusion enforced selective mobility for economic surplus when all other forms of cross-border mobility were put on hold.

The scope of this article is limited to a small group of migrants during a specific period. Nonetheless, the research raises future questions on differential inclusion through borders. First, what moments of mobility and immobility define unequal border crossings? Second, how and under what circumstances does the notion of border perception and differential inclusion change? And third, how are mobility practices and embodied experiences relevant to Schengen border policies? Future research should address these questions.

In sum, borders subjectively regulate, govern and restrict movement. Even during full border closure in March 2020, when it was officially declared that all freedom of movement should cease, borders remained selective, allowing continued passage based on the economic value of migrant labour. This insight is significant in an era of increasingly fortified EU external borders. Based on the narratives of the migrants interviewed in this study, the article contributes to the concept of differential inclusion in terms of how borders function in times of crisis and how the border regime of differential inclusion divides, adapts and channels mobilities.
